# Comparative genomic study on the complete plastomes of four officinal *Ardisia* species in China

**DOI:** 10.1038/s41598-021-01561-3

**Published:** 2021-11-15

**Authors:** Chunzhu Xie, Wenli An, Shanshan Liu, Yuying Huang, Zerui Yang, Ji Lin, Xiasheng Zheng

**Affiliations:** 1 Institute of Medicinal Plant Physiology and Ecology, School of Pharmaceutical Sciences, Guangzhou University of Chinese Medicine, 232th Waihuandong Road, Panyu District, Guangzhou, Guangdong China; 2 School of Pharmaceutical Sciences, Guangzhou University of Chinese Medicine, 232th Waihuandong Road, Panyu District, Guangzhou, Guangdong China

**Keywords:** Genetics, Molecular biology

## Abstract

*Ardisia* Sw. (Primulaceae) is naturally distributed in tropical and subtropical areas. Most of them possess edible and medicinal values and are popular in clinical and daily use in China. However, ambiguous species delineation and genetic information limit the development and utilization of this genus. In this study, the chloroplast genomes of four *Ardisia* species, namely *A. gigantifolia* Stapf, *A. crenata* Sims, *A. villosa* Roxb. and *A. mamillata* Hance, were sequenced, annotated, and analyzed comparatively. All the four chloroplast genomes possess a typical quadripartite structure, and each of the genomes is about 156 Kb in size. The structure and gene content of the *Ardisia* plastomes were conservative and showed low sequence divergence. Furthermore, we identified five mutation hotspots as candidate DNA barcodes for *Ardisia*, namely, *trnT-psbD*, *ndhF-rpl32*, *rpl32-ccsA*, *ccsA-ndhD* and *ycf1*. Phylogenetic analysis based on the whole-chloroplast genomes data showed that *Ardisia* was sister to *Tapeinosperma* Hook. f. In addition, the results revealed a great topological profile of *Ardisia’s* with strong support values, which matches their geographical distribution patterns. Summarily, our results provide useful information for investigations on taxonomic differences, molecular identification, and phylogenetic relationships of *Ardisia* plants.

## Introduction

The genus *Ardisia* Sw. belongs to the family Primulaceae. It consists of more than 700 species, which are typically found in tropical America, Pacific Islands, eastern Indian peninsula, and east to the south of Asia^1^. *Ardisia* plants are usually used as traditional medicine in China due to their multiple medicinal properties^2^, such as anti-neoplastic, anti-hypertension, anti-inflammatory, anti-arthritis, anti-angiogenesis, and analgesic. Therefore, it has become the focus of numerous researchers to explore the effective chemical components with various pharmacological effects in *Ardisia* species.^3–9^.

Researchers have investigated the genetic relationships among 24 *Ardisia* species by morphological characteristics and the *matK* genetic marker^10^. However, their inferences on the evolutionary relationship of *Ardisia* species were affected by unstable morphological characteristics or insufficient genetic information, resulting in significant differences in research results. The unclear phylogenetic relationships among *Ardisia* species has seriously hindered the further development of these important resources.

Chloroplasts are important photosynthetic organelles in green plants and have a set of genetic materials independent of the nucleus^11,12^. The chloroplast genome of angiosperms is generally considered to be a closed circular DNA molecule with a size between 120 and 160 kb and it has a typical conserved quadripartite structure, which consists of a large single copy (LSC) region, a small single copy (SSC) region and two copies of inverted repeats (IR)^13,14^. Furthermore, chloroplast DNA is maternally inherited, making them suitable for the analysis of phylogenetic relationships among species, especially for the closely related species^15,16^. With the application of high-throughput sequencing technology, it is now possible to reveal the phylogenetic relationships and develop molecular makers among species by using the genetic information of the whole chloroplast genome in many plant groups^17,18^. Therefore, we attempted to study the chloroplast genomes of *Ardisia crenata* Sims, *A. gigantifolia* Stapf, *A. mamillata* Hance and *A. villosa* Roxb. In order to elucidate the molecular evolution and genetic relationships of these species.

In this study, chloroplast genomes of four *Ardisia* species were sequenced. Firstly, we elucidated the size and structure of chloroplast genome. Secondly, the genomic repeats, and the variation of simple sequence repeats (SSRs) were identified. Finally, the phylogenetic relationships of the four *Ardisia* species and other Primulaceae species with available complete chloroplast genomes were analyzed. This work is of great significance for future research on the adaptive evolution of *Ardisia* species.

## Results

### Chloroplast genomes features of *Ardisia* species

The complete chloroplast genomes in the four *Ardisia* species ranged in size from 156,550 bp (*A. crenata*) to 156,734 bp (*A. mamillata*) (Table 1). All plastomes displayed typical quadripartite circle molecules consisting of a pair of IR regions (25,937–26,147 bp) separated by an LSC region (86,009–86,333 bp) and an SSC region (18,347–18,495 bp)*.* All four *Ardisia* species shared uniform overall GC contents, ranging from 37.1 to 37.3%. What’s more, the GC contents in the LSC, IR, SSC regions were 34.9–35.1%, 42.9–43.1% and 30.1–30.4%, respectively.

**Table 1:** Summary of the general features of four *Ardisia* chloroplast genomes.

Species	Total cp genome size (bp)	LSC region (bp)	IR region (bp)	SSC region (bp)	GC content (%)	GC content in LSC region (%)	GC content in IR region (%)	GC content in SSC region (%)
*A.gigamtifolia*	156,684	86,009	26,147	18,381	37.3	35.1	43.1	30.4
*A.crenata*	156,550	86,103	26,050	18,347	37.1	34.9	43.0	30.2
*A.mamillata*	156,734	86,333	25,999	18,403	37.1	35.0	42.9	30.2
*A.villosa*	156,645	86,276	25,937	18,495	37.1	35.0	43.1	30.1

**Figure 1:**
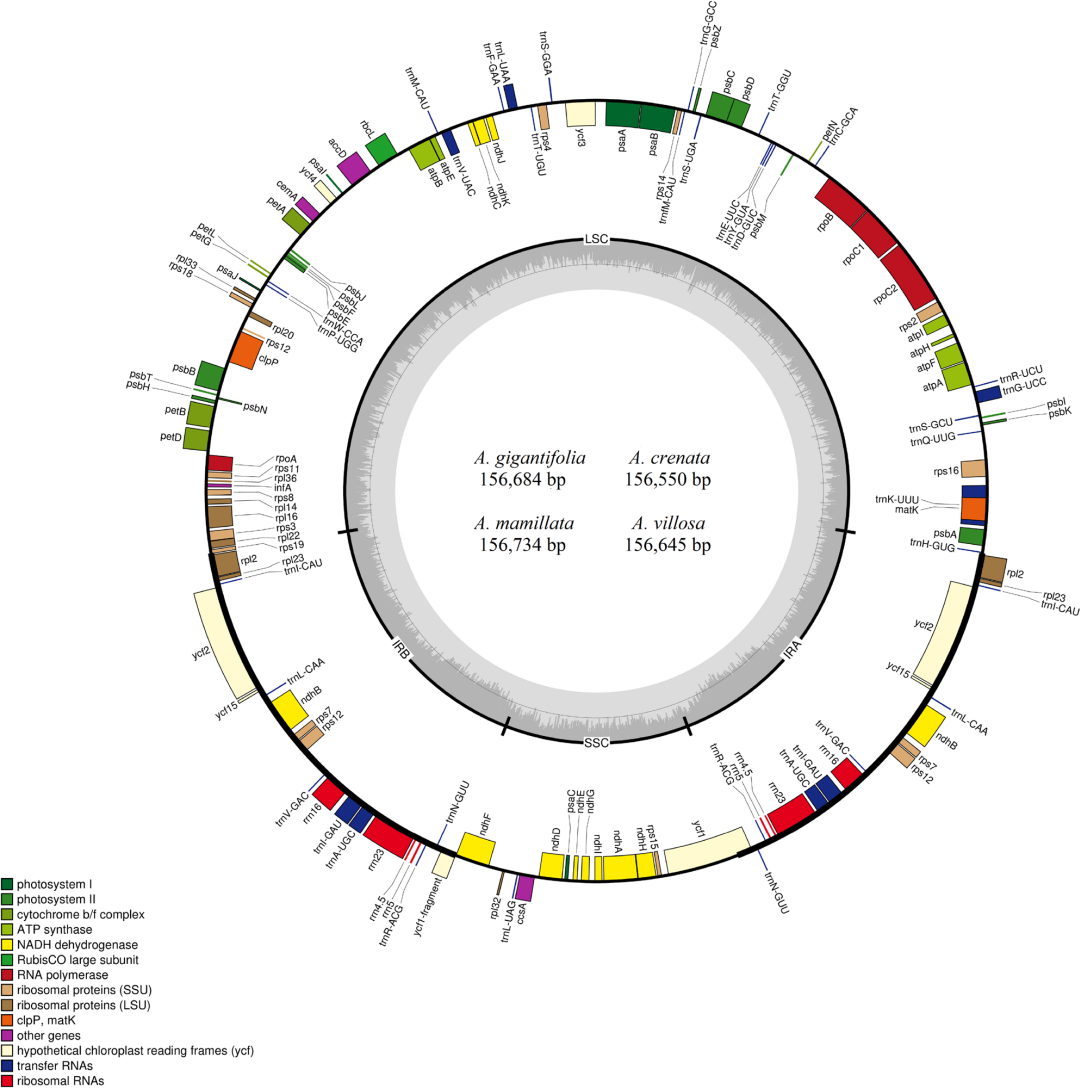
Plastome features of *Ardisias*. Genes exhibited inside the circle are transcribed in a clockwise direction and those outside are transcribed counterclockwise. The gray arrow represents gene direction. Different genes are color-coded. The dashed gray area in the inner circle shows the percent GC content of genes, whereas the lighter gray corresponds to AT content.

After annotation, we identified a total of 114 unique genes, including 80 protein-coding genes (CDS), 30 tRNAs and 4 four rRNAs (Table 2, Table S2). Out of these, 19 genes were duplicated in the IR regions, including seven tRNAs, four rRNAs (*rrn16*, *rrn23*, *rrn4.5* and *rrn5*) and eight protein-coding genes (Fig. 1). The GC content of the first, second and third codon sites in the CDS regions of these four *Ardisia* chloroplast genomes were 40.3%/34.6%/38.1% (*A. gigamtifolia*), 32.3%/41.6%/38.6% (*A. crenata*), 42.7%/38.5%/31.3% (*A. mamillata*), 38.1%/31.3%/43.3%(*A. villosa*), respectively (Table S2). Furthermore, there were seventeen genes that harbored introns, among which fifteen distinct genes contain only a single intron, whereas two genes (*ycf3*, *clpP*) harbored two introns. The *trnK-UUU* gene owned the longest intron (2534–2550 bp) and its intron region contained the *matK* gene (Table S3). Across these chloroplast genomes, the *rps12* was found to trans-splice in the IR-LSC region in which the 3’-end is duplicated in the IR regions and the 5’-end located in the LSC region.

**Table 2:** Statistics on the number of genes and nucleotide positions in the chloroplast genomes of four *Ardisia* species.

Species	Number of				CDS (bp)	ATCG contents in CDS (%)				ATCG contents in 1st position (%)				ATCG contents in 2nd position (%)				ATCG contents in 3rd (%)			
	Unique genes	Protein-coding genes	tRNAs	rRNAs		A	T	C	G	A	T	C	G	A	T	C	G	A	T	C	G
*A.gigamtifolia*	114	80	30	4	79,451	30.6	31.7	17.7	20.0	30.7	29.0	18.8	21.5	29.8	36.0	17.5	17.1	31.4	31.0	16.7	21.4
*A.crenata*	114	80	30	4	79,613	30.7	31.8	17.6	19.9	31.9	36.0	15.3	17.0	29.8	29.0	17.8	23.8	30.4	31.0	19.6	19.0
*A.mamillata*	114	80	30	4	79,603	30.7	31.8	17.6	19.9	31.3	26.0	18.0	24.7	29.6	32.0	19.6	18.9	31.3	37.0	15.1	16.2
*A.villosa*	114	80	30	4	79,550	30.7	31.8	17.6	20.0	29.8	32.0	19.5	18.6	31.1	38.0	15.1	16.2	31.2	25.0	18.2	25.1

### Codon usage

**Figure 2:**
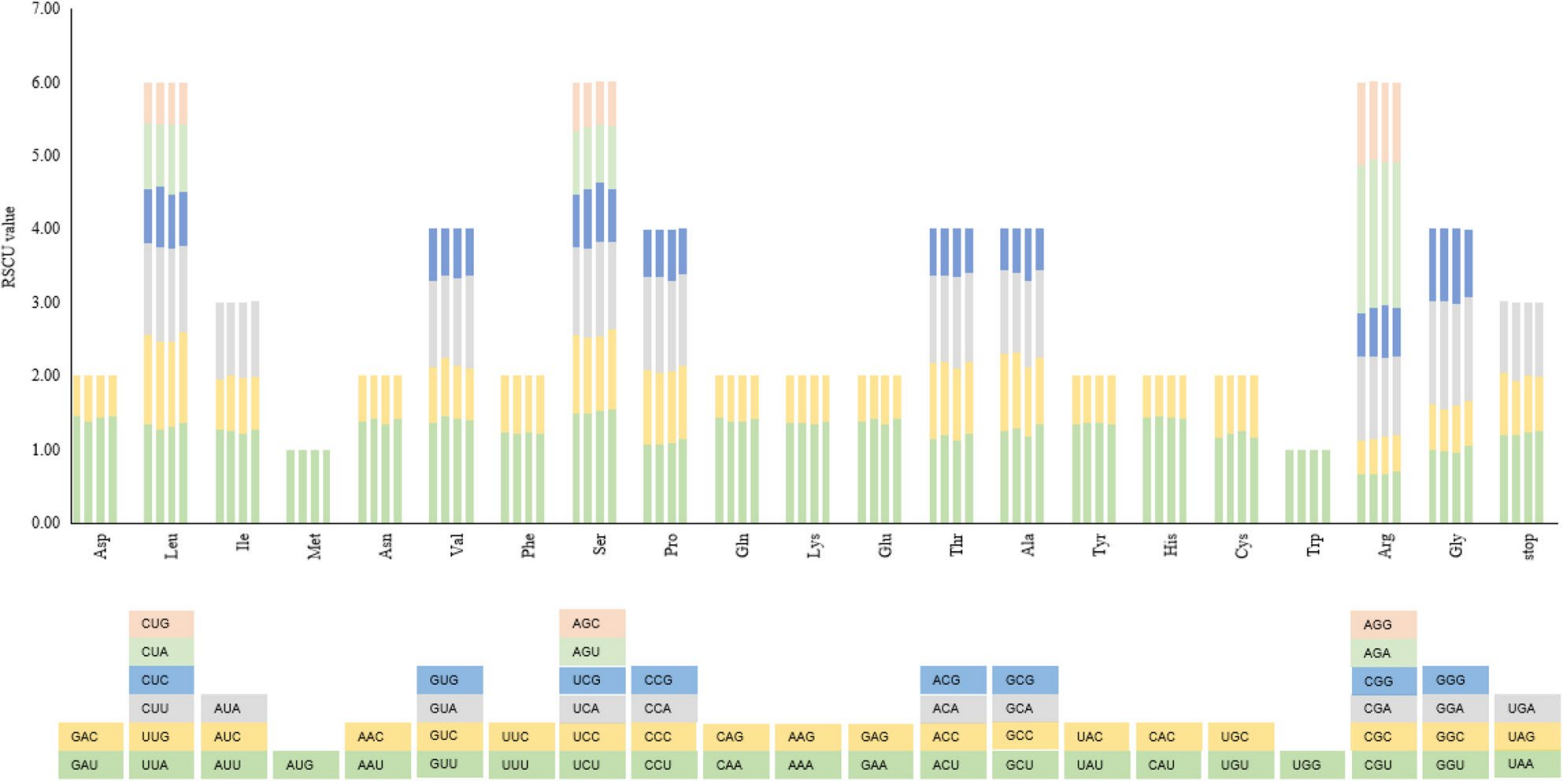
Codon content for protein-coding sequences in four *Ardisia* chloroplast genomes. The x-coordinate represents the 20 amino acids and the terminators, while the y-coordinate represents the RSCU value of the corresponding amino acid of each species. The species of each amino acid from left to right are *A. gigantifolia*, *A. crenata*, *A. mamillata* and *A. villosa*, respectively. The different colors of each amino acid corresponding to the codon of the same color below.* Asp* asparticacid, *Leu* leucine, *Ile* isoleucine, *Met* methionine, *Asn* asparagine, *Val* valine, *Phe* phenylalanine, *Ser* serine, *Pro* proline, *Gln* glutarnine, *Lys* lysine, *Glu* glutamic acid, *Thr* threonine, *Ala* alanine, *Tyr* tyrosine, *His* histidine, *Cys* cysteine, *Trp* tryptophan, *Arg* arginine, *Gly* glycine.

In these *Ardisia* chloroplast genomes, the protein-coding genes presented a total of 52,183 to 52,244 codons, with the *A. mamillata* containing the most abundant codons and the *A. crenata* containing the least (Table S4). The most frequently used codon in the four plastomes was the UUU that encoded phenylalanine (Phe), while the least-used codon was the GCG encoded alanine (Ala). Among the four species of *Ardisia*, the number of codons with RSCU > 1 was equally and the RSCU values of the same codons in our four plastomes were slightly different (Fig. 2, Table S4). The results of RSCU in the four *Ardisia* species showed A or T was biased toward a higher nucleotide frequency than C or G at the third codon position and the result was similar to other angiosperms chloroplast genomes.

### RNA editing analysis

We used the PREP-Cp database^19^ to predict possible RNA editing sites in the chloroplast genomes of four *Ardisia* species. The results revealed that (Table S5) among the 80 protein-coding genes in the chloroplast genomes of the four species, RNA editing occurred in 14 genes in the *A. gigantifolia* and 15 genes occurred in the other three species, containing 47–50 editing sites. All editing events involved C to U conversion and also caused changes in amino acids. A statistical analysis of the codon locations showed that 17 mutations occurred in the first position of the codon, while the remaining were found in the second position. There were eleven types of amino acid transformation, including T → I, P → S, P → L, S → L, A → V, H → Y, L → F, S → F, T → M, R → W and R → C, among which the S → L transformation was the most common. Among all genes undergoing editing, *rpoC2* and *rpoB* owned the most editing sites (7–10), followed by *ndhB* (5).

### Repeat and simple sequence repeat (SSR) analysis

**Figure 3:**
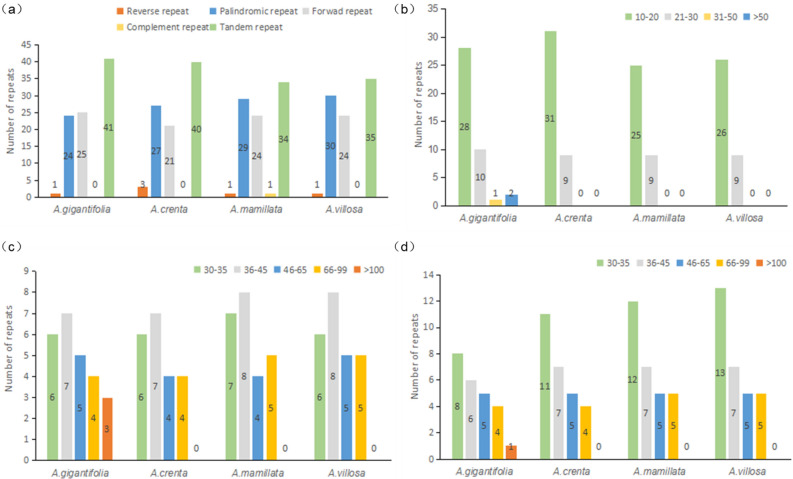
Repeat analysis of four *Ardisias*. The ordinates represent the number of **(a)** different repeat types, **(b)** tandem repeats, **(c)** forward repeats and **(d)** palindromic repeats in four Ardisia chloroplast genomes, respectively. Repeats with different lengths are indicated in different colors, the ordinate represents the number of repeats.

Using PREter and Tandem online tools, the repetitive sequences of four *Ardisia* plastomes were analyzed, and the quantitative comparison maps of reverse (R), forward (F), palindromic (P), complement (C) and tandem(T) repeat sequences were summarized (Fig. 3).

As shown in Fig. 3a, there was no significant difference in the number of repeated sequences among the four plants, with the number of 50–55 long repeats and 34–41 tandem repeats. It should be noted that the number of F and P duplications in each species was about 1:1, while no C duplications were observed in any of the three species except for *A. mamillata*.

**Figure 4:**
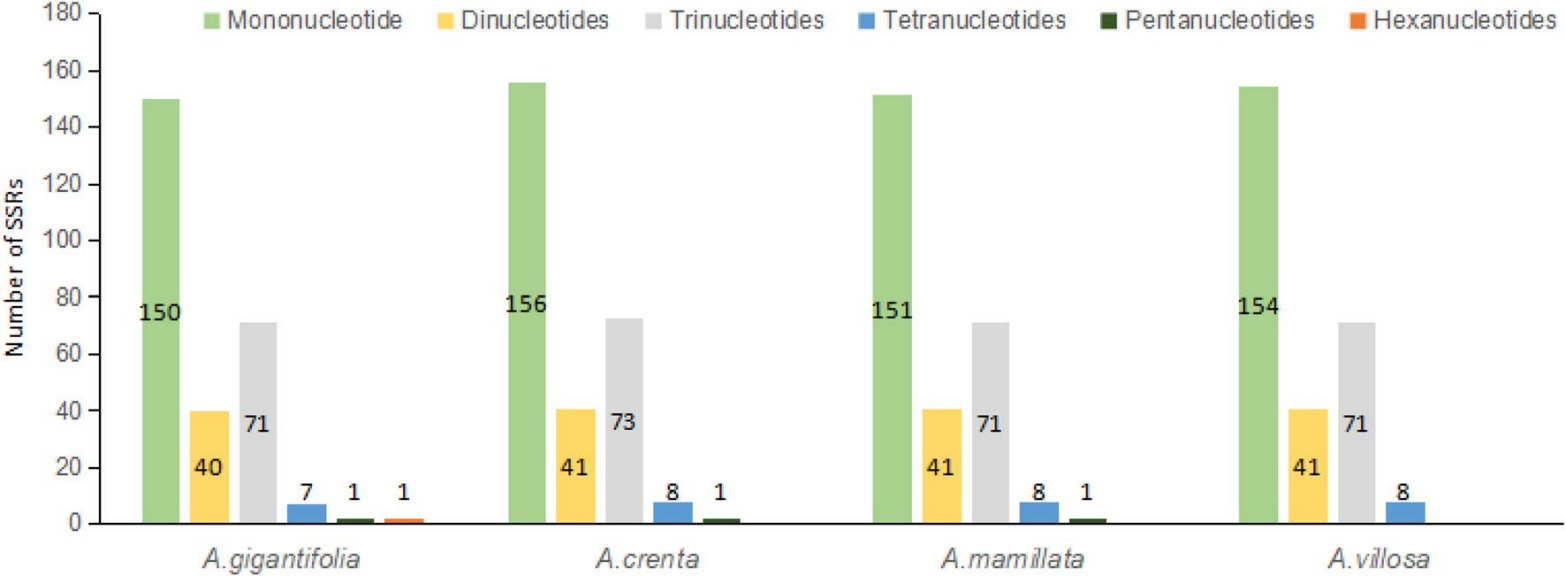
SSR loci analysis of four *Ardisias*.

Simple sequences repeats (SSRs), the DNA sequences consisting of multiple repeats of 1–6 nucleotide(s), are widely distributed in eukaryotes. Using MISA analysis, six types (i.e., mono-, di-, tri-, tetra-, penta-, and hexa-nucleotides) SSRs were detected in four plastomes of *Ardidia* species, and each chloroplast genome was found to contain 270 (*A. gigantifolia*) to 279 (*A. crenata*) SSRs. *A.crenta* and* A. mamillata* contained five types of SSRs, excluding the hexanucleotide, while the *A. gigantifolla* and  *A.villosa* contained six and four types, respectively. (Fig. 4, Table S6). For the different unit size, mononucleotide SSRs were most highly abundant (55.5%, 55.9%, 55.5%, 56.2% in *A. gigantifolla, A. crenata, A. mamillata* and *A. villosa,* respectively).

In conclusion, the comparative analysis of the repeats and SSRs in the four *Ardisia* chloroplast genomes showed that (Figs. 3, 4). *A. crenata*, *A. mamillata* and *A.villosa* were resembling with each other, while the repeats and SSR types of the *A. gigantifolia* were more than those of the other chloroplast genomes. Probably because the *A. gigantifolia* belongs to the serrate group and the other three plants belong to the crenate group of the *Ardisia* genus, this indicated that there were differences between different groups of species.

### Comparative analysis of the *Ardisia* plastomes

**Figure 5:**
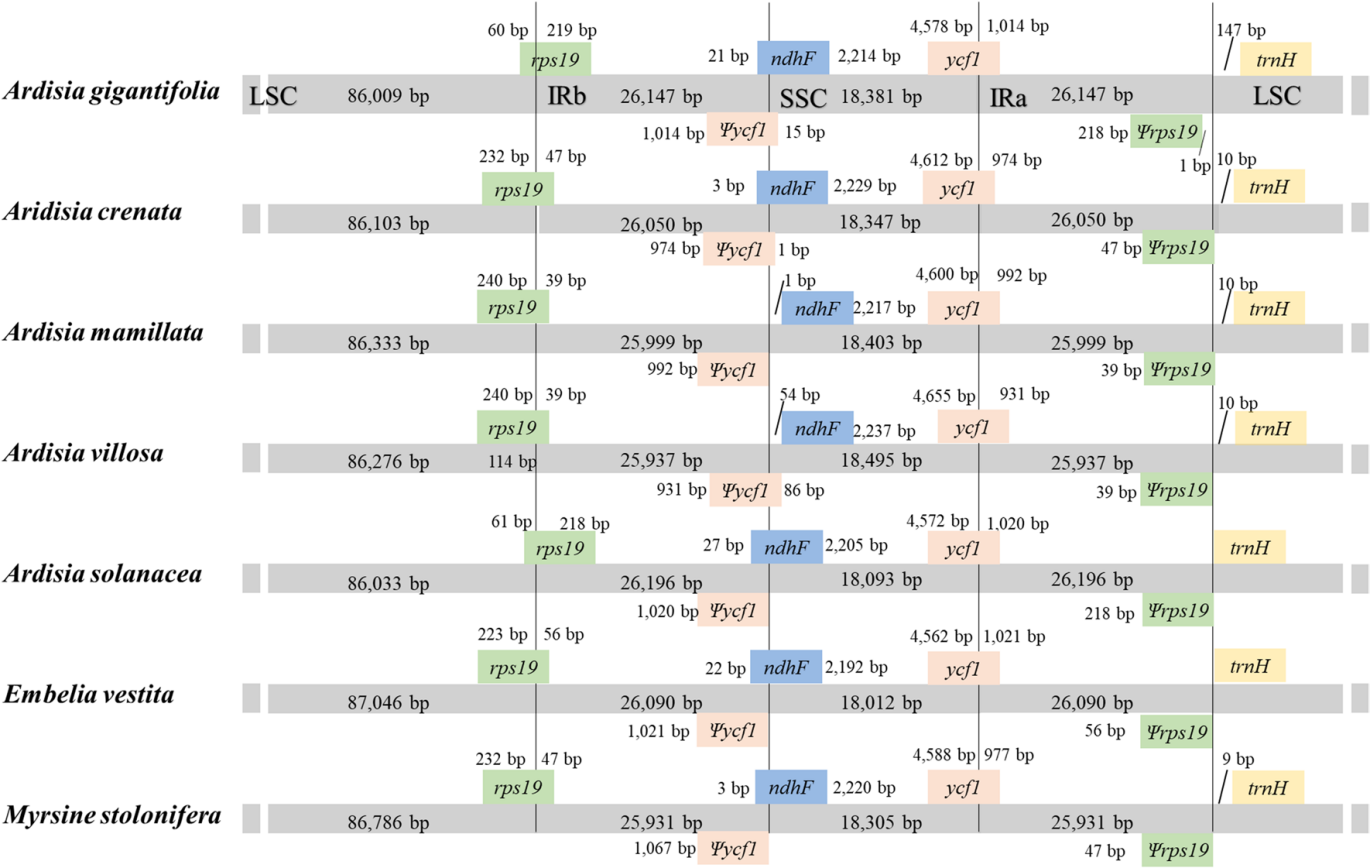
Comparison of the IRs, LSC, SSC boundary features among the seven Primulaceae chloroplast genomes. The number represents the distance between the ends of the genes and the boundaries. Ψ: pseudogenes.

As mentioned above, the typical quadripartite structure of the chloroplast genome consists of two different single-copy regions and two inverted repeat regions. In order to get a better understanding of the IR region evolution, a comparative study was performed between the four *Ardisia* chloroplast genomes and several related species, including *Ardisia solanacea* (Poir.) Roxb., *Embelia vestita* Roxb.*,* and *Myrsine stolonifera* (Koidz.) E. Walker, investigating the length of the IR region and the variation between the IR regions and SC (LSC and SSC) boundaries. In general, the boundaries between the IRs and LSC/SSC regions of seven Primulaceae species exhibit a similar pattern, in which the SSC/IRa node was located in the *ycf1* gene and the LSC/IRb position was located in the *rps19* gene. Consequently, due to this cross-boundaries phenomenon, corresponding incomplete copies of *ycf1* and *rps19* appear at the boundaries of IRb/SSC and IRa/LSC junction, respectively (*ψycf1*, *ψrps19*). Meanwhile, the *ndhF* gene was located at the junction of IRb and SSC, and its 3' end was overlapped with *ψycf1* in the other six plants with the exception of *A. mamillata*. The *trnH* gene was present at the junction of IRa/LSC (Fig. 5).

**Figure 6:**
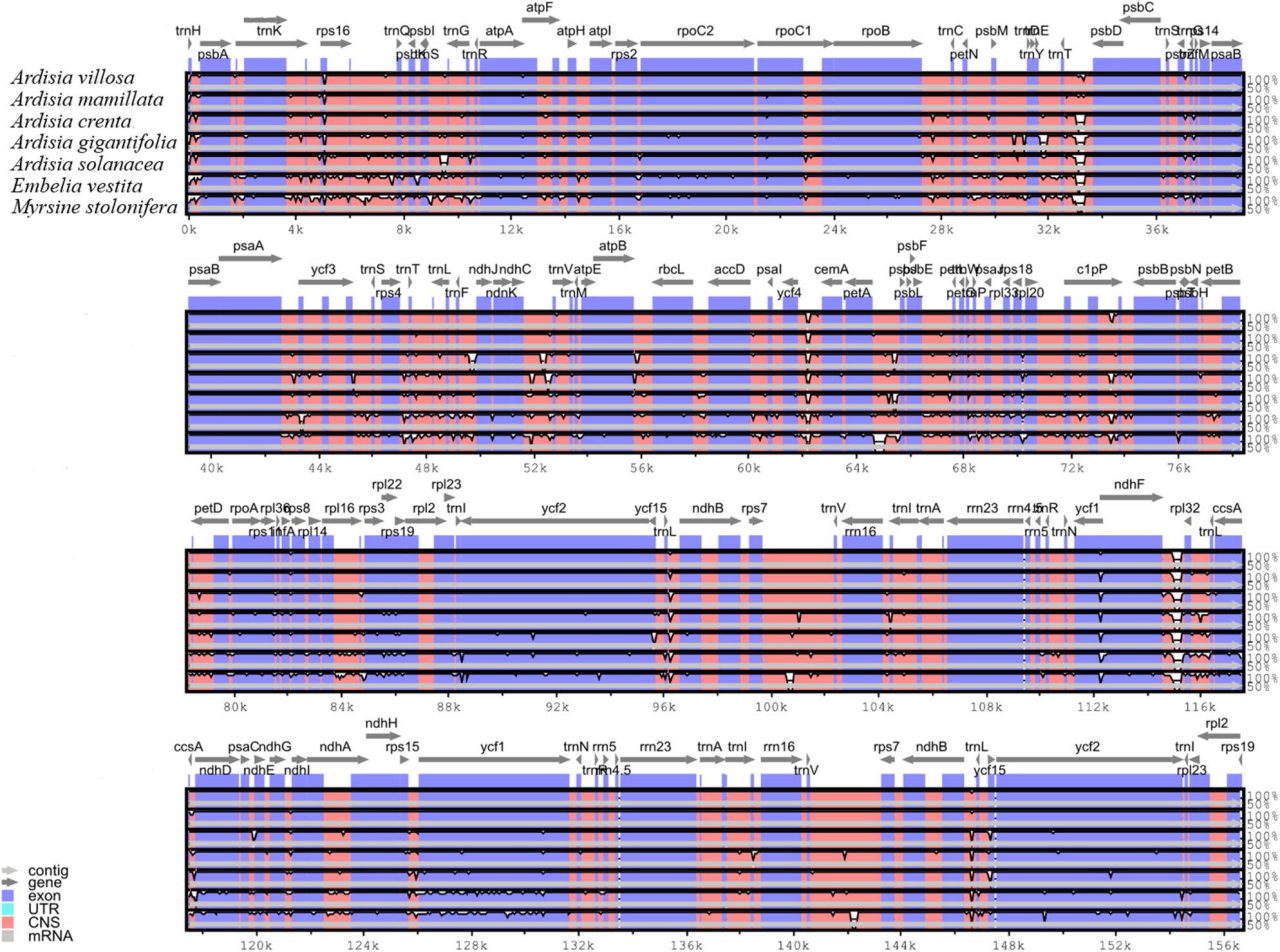
Overall sequence identity of plastomes belonged to seven Primulaceae species. Gray arrows indicate the transcriptional direction of genes, purple bars represent coding regions, and pink bars represent non-coding regions. The vertical scales represent percentage similarity ranging from 50 to 100%.

The mVISTA platform was conducted for comparing the overall identity among the four *Ardisia* chloroplast genomes and three other reported Primulaceae species. As illustrated in Fig. 6, all seven species had similar chloroplast gene sequence and structure, and the non-coding regions showed more variation than the coding regions as colored in purple bars. It is noteworthy that the two IR regions were more conservative than the remaining two regions. Further study found that the gene spacer regions were significantly different among the chloroplast genomes of the seven Primulaceaes, for example, *trnT-trnL*, *trnT*-*psbD*, *rpl32-ccsA, ycf1*, *ndhF*-*rpl32* and *ccsA*-*ndhD*.

To explore the sequence differences among *Ardisia* chloroplast genomes, the number of nucleotide substitutions were counted and genetic distances were calculated based on the Kimura-2-parameter through the MEGA tool (Table 3). The results showed that the number of nucleotide substitutions of the four species was 171–1237 and the genetic distance was 0.001050–0.007980. In general, the number of nucleic variations in the *A. gigantifolia* chloroplast genome sequence was higher than that of *A. crenata*, *A. mamillata*, and *A. villosa*. Similarly, the genetic distance was greater. It confirmed the species diversity among different groups of the *Ardisia* genus.

**Table 3:** Genetic distance analysis of four *Ardisia* chloroplast genomes.

Species	*A. mamillata*	*A. villosa*	*A. gigantifolia*	*A. crenata*
*A. mamillata*		171	1157	444
*A. villosa*	0.001050		1237	515
*A. gigantifolia*	0.007463	0.007980		1,074
*A. crenata*	0.002835	0.003265	0.006913	

The upper triangle indicates the number of amino acid substitutions between pairs of sequences, and the lower triangle indicates the genetic distance of Kimura-2-parameter.

**Figure 7:**
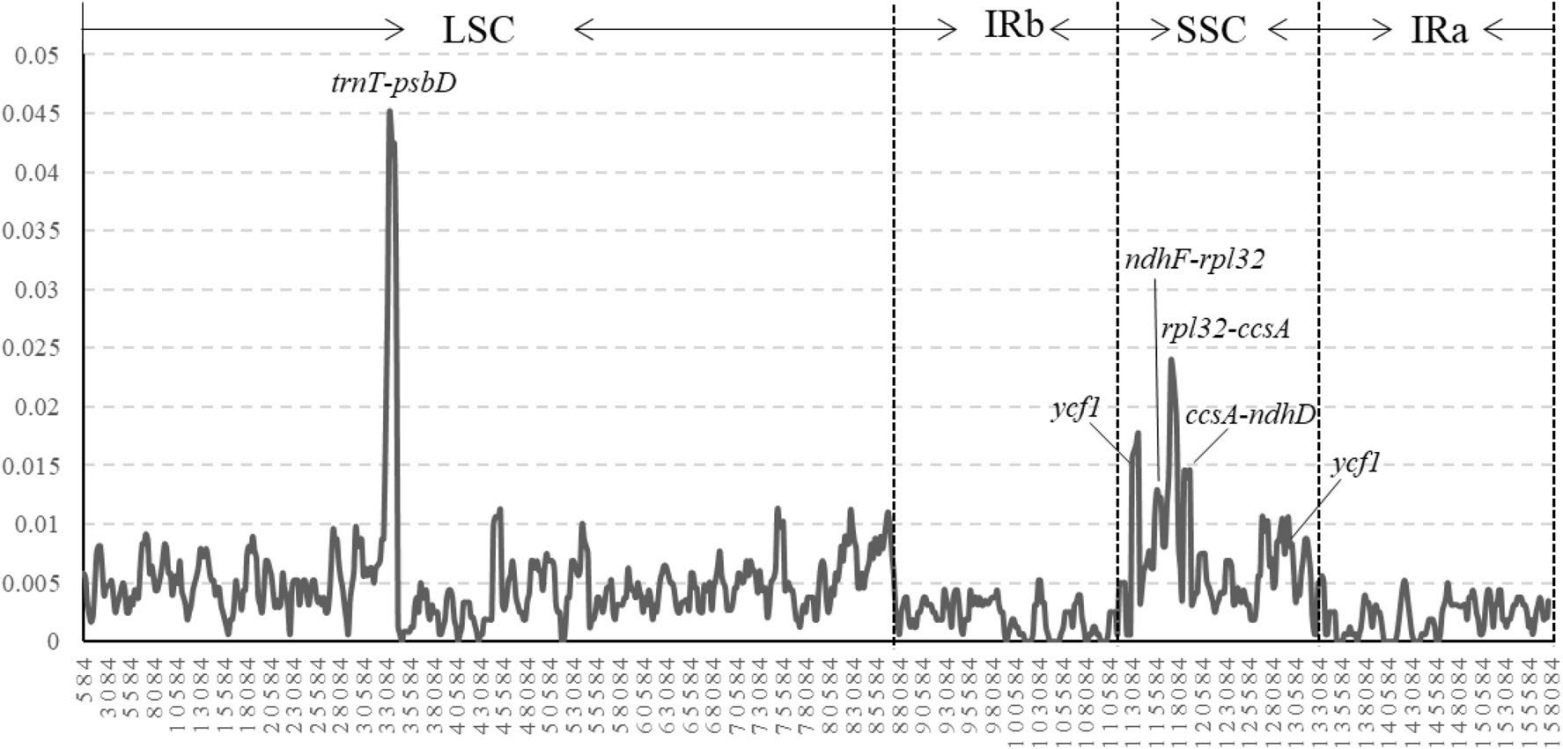
Comparative analysis of the nucleotide variability by Pi values of the four *Ardisia* CP genomes presented in a sliding window (window length: 800 bp; step size: 200 bp). The X-axis represents the position of the point in the window, while the Y-axis represents the nucleotide polymorphism in each window.

We further calculated the nucleotide polymorphisms of 800 bp window. Among the four *Ardisia* chloroplast genomes, the Pi values varied from 0 to 0.045 and detected five higher-variable regions (Pi > 0.012), namely *trnT-psbD*, *ndhF-rpl32*, *rpl32-ccsA*, *ccsA-ndhD* and *ycf1* (Fig. 7)*.*

### Phylogenetic analysis

**Figure 8:**
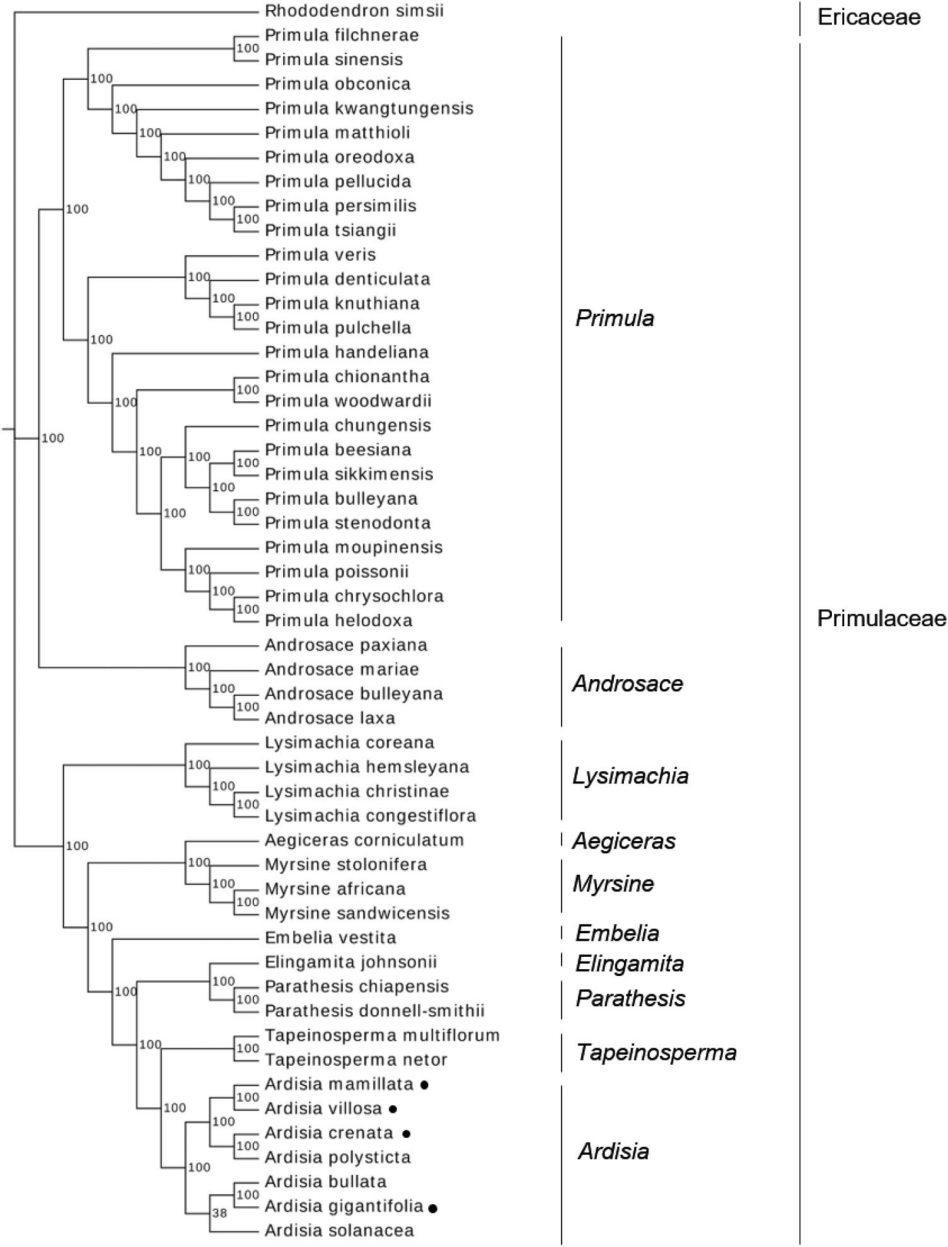
Phylogenetic tree reconstruction of 51 plants using maximum likelihood methods based complete chloroplast genomes. The bootstrap values were labeled on the evolutionary branches and four species in this study were labeled with solid black dots.

We downloaded 46 published chloroplast genome sequences of Primulaceaes from the NCBI database and established an ML tree with four *Ardisia* chloroplast genomes in this study to clarify better the evolutionary relationships within the Primulaceae (Fig. 8). The *Rhododendron simsii* Planch. is an extraneous group of Ericaceae, and the NCBI accession numbers of 47 species are listed in Table S1. It could be seen from Fig. 8 that among the genera of Primulaceae, *Tapeinosperma* was observed to be a sister lineage of *Ardisia* with strong bootstrap values of 100%. In addition, The *A. mamillata*, *A. villosa*, *A. polysticta*, and *A. crenata* which belong to the crispardisia group from the *Ardisia* were well clustered and could therefore be distinguished from *A. gigantifolia* of the bladhia group.

## Discussion

The results indicated that the chloroplast genomes of four *Ardisia* species were similar in structure, genome length, and organization. The length of the four chloroplast genomes ranged from 156,550 bp (*A. crenata*) to 156,734 bp (*A. mamillata*), it was within the size range of chloroplast genomes in other angiosperms^20,21^. The *Ardisia* chloroplast genomes displayed the typical quadripartite structure with similar GC content, indicating that the almost identical levels among the *Ardisia* chloroplast genomes. The IR regions had the highest GC content, which might be caused by the decrease of AT nucleotides in the four rRNA genes (*rrn16, rrn23, rrn4.5, rrn5*)^22^. Compared to the previously published data, the structural features of the *Ardisia* chloroplast genomes were highly similar to those of other Primulaceae chloroplast genomes^23^.

Introns, a group of self-catalytic ribozymes that could splice their own excision from mRNA, tRNA, and rRNA precursors, help to infer phylogenetic relationships. The length of exons and introns in genes was important information in plant chloroplast genome^24,25^. In this study, there were two genes (*ycf3* and *clpP*) including two introns in four *Ardisia* chloroplast genome. The *ycf3* has been reported to be a gene closely related to photosynthesis^26^. Therefore, the acquisition of *ycf3* gene will make an important contribution to the further study of the *Ardisia* chloroplast.

The synonymous codons usually only mutate in the third position to adapt to the existence of gene mutations and natural selections^27^. The relative synonymous codon usages (RSCU) refers to the frequency of specific codons in synonymous codons for a certain amino acid^28,29^. The above results showed that the codon preference of the four *Ardisia* species is high consistency, which is congruent with other genera.

After being transcribed, chloroplast mRNA molecule usually undergoes RNA editing, a process of C-to-U conversion is performed at specific sites to regulate gene expression and translation in the chloroplast. RNA editing plays an important regulatory role in plant growth, development, stress response, and other physiological and biochemical processes^30^. Identification of RNA editing sites will benefit the study of related biological functions. In our work, potential RNA editing sites were identified in 14–15 protein-coding genes of four *Ardisia* species. All editing events involved C to U conversion and also caused changes in amino acids^31,32^, while the S → L transformation was the most common. It was similar to the composition characteristics of RNA editing in chloroplast genomes of other plants.

Repeat sequences detected in plastomes have been proven to be correlated with rearrangement, sequence divergence, and recombination^33^. They provide vital information for understanding the evolutionary history and sequence divergence of plant species^34,35^. With the advantages of high polymorphism, stability and repeatability, SSRs have been widely used in genetic diversity analysis, species identification, and molecular breeding^36–38^. We detected six types of SSRs in four plastomes of *Ardidia* species. According to the result, these high variabilities of SSRs may provide strong value and evidence for molecular breeding and identification of medicinal plants.

IR regions are the most conserved regions in the chloroplast genomes^39^. Frequent expansions and contractions at the junctions of SSR and LSC with IRs have been recognized as evolutionary signals for which illustrating the relationships among taxa^40^. It is believed that the contractions and expansions of IR regions in angiosperms are generally accompanied by the changes in the length and distribution of *ycf1* and *rps19*^41^. We found that the boundaries between the IRs and LSC/SSC regions of seven Primulaceae species exhibit a similar pattern, in which the *ycf1* gene and the *rps19* gene were located in the SSC/IRa node and the LSC/IRb node respectively.

Studies have shown that mutations in chloroplast genomes can be concentrated and become hotspots for identification and defined as DNA barcoding^42,43^. It is a remarkable fact that single copy regions’ mutation rate is significantly higher than reverse repeat regions. Among the four *Ardisia* chloroplasts, five higher-variable regions (*trnT-psbD*, *ndhF-rpl32*, *rpl32-ccsA*, *ccsA-ndhD* and *ycf1)* were detected, which can be selected as the DNA barcoding of *Ardisia*.

In recent years, the chloroplast genome has become an indispensable tool to investigate phylogenetic development of species, and it could be widely used in phylogenetic reconstruction of plants at different taxonomic levels, such as order, family, genus and species^44,45^. The genetic relationships of *Ardisia* in Primulaceae are still somewhat uncertain. Phylogenetic relationships of Primulaceae species were inferred based on the available plastomes using ML methods. As indicated by Fig. 8, all branches of the phylogenetic tree are strongly supported. The phylogenetic analyses revealed that four chloroplast genomes presented a close relationship with other reported *Ardisias,* and different groups of *Ardisia* could be distinguished from each other (crispardisia group and bladhia group), which clearly indicated that these phylogenetic results are consistent with morphological taxa. Hence, in order to better elucidate the phylogenetic relationships of Primulaceae, more chloroplast genomes are needed to be sequenced.

## Conclusions

The complete chloroplast genomes of *A. crenata*, *A. gigantifolia*, *A. villosa*, and *A. mamillata* were sequenced and characterized. These four plastomes were presented as circular molecular with typical quadripartite structure and shared a similar gene composition and the base content. In the *Ardisia* chloroplast genomes, mononucleotide SSR and tandem repeats were dominant. Through the analysis of the variable sites, five potential mutation hotspots were found, which laid the foundation for the molecular identification of this genus. Furthermore, four chloroplast genomes presented a close relationship with other reported *Ardisia* species and confirmed the sister linage with *Tapeinosperma*. Interestingly, different groups of *Ardisia* could be distinguished from each other. In a nutshell, these highly variable sites and the complete chloroplast genomes provided sufficient information for contributing to the further study of the molecular evolution and genetic relationship among Primulaceae.

## Methods

### Plant materials and DNA extraction

Four *Ardisia* species used in this experiment were all collected from the medicinal botanical garden of Guangzhou University of Chinese Medicine (GUCM). These species have not been included in the list of national key protected plants, and permission was not necessary for collecting them. Experimental research on plants, including the collection of plant materials, complies with relevant institutional, national, and international guidelines and legislation. The authenticity of the plant materials has been verified by Professor Ji Lin and Dr. Guifang Zhang of GUCM. Voucher specimens of *A. crenata* (voucher number 441823LY1019), *A. gigantifolia* (441823LY0880), *A. mamillata* (441823LY0451), and *A. villosa* (441823LY0951) were deposited at the Chinese medicine herbarium of GUCM.

Fresh young leaves of *A. crenata*, *A. gigantifolia*, *A. mamillata,* and *A. villosa* were obtained, and be conducted to DNA extraction with the Plant Genomic DNA Kit (Tiangen, China). Then, the DNA concentration and quality of each sample were checked by Ultraviolet Spectroscopy with a Nanodrop-2000 spectrometer (Nanodrop Technologies, Wilmington, DE, USA) and agarose gel electrophoresis.

### Genome sequencing, assembly and annotation

Approximately 3–5 µg of total DNA was sheared into short-insert (350 bp) fragments, followed by library construction. Then, these libraries were evaluated and conducted to genome sequencing with an Illumina HiSeq 4000 platform, generating approximately 5 GB of raw data for each sample.

Clean reads were retained after filtering the low-quality reads and removing adapters using the Trimmomatic (v0.39, Max Planck Institute of Molecular Plant Physiology, Potsdam, Germany). Cp-like reads were extracted from clean Reads according to sequence similarity using the bwa software (v0.7.17)^46^, then these cp-like reads were assembled using the SPAdes (v3.13.1) to generate contigs. These contigs were plotted against reference plastome to adjust their orientation and gaps were filled with the GapCloser^47^. All clean data was then mapped to the final assembly sequence and visualized by the IGV tool to get an overview of the reads coverage, resulting in complete chloroplast genomes.

Preliminarily gene annotation of four complete chloroplast genomes was performed by the GeSeq online tool (https://chlorobox.mpimp-golm.mpg.de/geseq.html) with default parameters^48^. and further revised manually based on the referential chloroplast genome of *A.solanacea* (NC_045098.1)*.* Finally, a gene map of the annotated *Ardisia* chloroplast genome was drew using the OGDRAW tool (http://ogdraw.mpimp-golm.mpg.de/)^49^.

### Codon usage and prediction of RNA editing sites

To examine the deviation in synonymous codon usage, the amino acid frequency and the relative synonymous codon usage (RSCU) were analyzed using the Molecular Evolutionary Genetic Analysis (MEGA, version 7)^50^. To predict the possible RNA editing sites in the four *Ardisia* chloroplast genomes, the online tool of Predictive RNA Editor for Plants (PREP, http://prep.unl.edu/)^19^ was adapted with a cutoff value set as 0.8.

### Analysis of repeat elements in four *Ardisias*

REPuter (https://bibiserv2.cebitec.uni-Bielefeld.de/reputer)^51^ was used to identify long repeat sequences with a Hamming distance set as three and a minimum repeat size set as 30 bp. Additional, Tandem repeats finder (https://tandem.bu.edu/trf/trf.html)^52^ was used to detect tandem repeats and the SSRs in the *Ardisia* chloroplast genomes were identified using MISA software^53^, with alignment parameters set to 2, 7 and 7 for matches, mismatches and indels.

### Comparative analysis

The mVISTA tool (http://genome.lbl.gov/vista/index.shtml) was used to investigate the divergences between the *Ardisia* complete chloroplast genomes and three referential Primulaceae species in the Shuffle-LAGAN mode. Moreover, IR expansions/contractions were summarized manually^54^.

Those selected chloroplast genomes were aligned using the MAFFT (v7.419) software with a default setting and then adjusted manually by Se-Al 2.024^55^. Next, MEGA7 was used to calculate the single nucleotide variants (SNV) and the mean genetic distance between the chloroplast genome sequence of *Ardisias*. Additionally, DnaSP v5.10 was used to calculate the Pi value and the SNP variation sites of the four *Ardisia* chloroplast genomes. The step size was set to 200 bp with an 800 bp window length^56^.

### Phylogenetic analysis

A phylogenetic analysis was conducted using the complete chloroplast genomes of four *Ardisia* species and forty-six Primulaceae species, with an outgroup was *Rhododendron simsii* which belongs to Ericaceae. These complete chloroplast genomes were downloaded from the NCBI database (Table S1) and multi-sequence alignment was performed by the MAFFT program. Finally, the phylogenetic analyses with Maximum likelihood (ML) was conducted using the GTR + G substitution model, which was selected based on model screening. Bootstrap analysis was executed with 1,000 replicates and TBR branch swapping.

## Supplementary Information

Supplementary Tables.

## Data Availability

The sequencing datasets generated during the current study are available at China National GeneBank with project number as CNP0001336 (https://db.cngb.org/search/project/CNP0001336/). The accession numbers of four species are CNP0001336 (*Ardisia gigantifolia*), CNS0285138 (*Ardisia crenata*), CNS0285139 (*Ardisia mamillata*), CNS0285140 (*Ardisia villosa*).

## References

[1] Kobayashi H, de Mejia E (2005). The genus *Ardisia*: A novel source of health-promoting compounds and phytopharmaceuticals. J. Ethnopharmacol..

[2] Hu J, Liu S, Cheng Q, Pu S, Mao M, Mu Y, Dan F, Yang J, Ma M (2020). Novel method for improving ardicrenin content in hairy roots of *Ardisia crenata* Sims plants. J. Biotechnol..

[3] Hamid RA, Fong LM, Ting YL (2017). Anti-arthritic and gastroprotective activities of *Ardisia crispa* root partially mediated via its antioxidant effect. J. Complement. Integr. Med..

[4] Jasamai M, Jalil J, Jantan I (2015). Molecular docking study on platelet-activating factor antagonistic activity of bioactive compounds isolated from Guttiferae and *Ardisia* species. Nat. Prod. Res..

[5] Lee IS, Cho DH, Kim KS, Kim KH, Park J, Kim Y, Jung JH, Kim K, Jung HJ, Jang HJ (2018). Anti-inflammatory effects of embelin in A549 cells and human asthmatic airway epithelial tissues. Immunopharmacol. Immunotoxicol..

[6] Mu LH, Bai L, Dong XZ, Yan FQ, Guo DH, Zheng XL, Liu P (2014). Antitumor activity of triterpenoid saponin-rich *Adisia gigantifolia* extract on human breast adenocarcinoma cells in vitro and in vivo. Biol. Pharm. Bull..

[7] Mu LH, Wang LH, Wang YN, Liu P, Yan C (2020). Antiangiogenic effects of AG36, a triterpenoid saponin from *Ardisia gigantifolia* stapf. J. Nat. Med..

[8] Yao C, Jin CL, Oh JH, Oh IG, Park CH, Chung JH (2015). *Ardisia crenata* extract stimulates melanogenesis in B16F10 melanoma cells through inhibiting ERK1/2 and Akt activation. Mol. Med. Rep..

[9] Shahinozzaman M, Ishii T, Halim MA, Hossain MA, Islam MT, Tawata S (2019). Cytotoxic and anti-inflammatory resorcinol and alkylbenzoquinone derivatives from the leaves of *Ardisia sieboldii*. Z. Naturforsch. C. J. Biosci..

[10] Liu Y, Wang K, Liu Z, Luo K, Chen S, Chen K (2013). Identification of medical plants of 24 *Ardisia* species from China using the matK genetic marker. Pharmacogn. Mag..

[11] Duan S, Lu B, Li Z, Tong J, Kong J, Yao W, Li S, Zhu Y (2007). Phylogenetic analysis of AA-genome *Oryza* species (Poaceae) based on chloroplast, mitochondrial, and nuclear DNA sequences. Biochem. Genet..

[12] Marchand J, Heydarizadeh P, Schoefs B, Spetea C (2018). Ion and metabolite transport in the chloroplast of algae: Lessons from land plants. Cell Mol. Life Sci..

[13] Cui Y, Chen X, Nie L, Sun W, Hu H, Lin Y, Li H, Zheng X, Song J, Yao H (2019). Comparison and phylogenetic analysis of chloroplast genomes of three medicinal and edible amomum species. Int. J. Mol. Sci..

[14] Yang J, Yue M, Niu C, Ma XF, Li ZH (2017). Comparative analysis of the complete chloroplast genome of four endangered herbals of Notopterygium. Genes (Basel).

[15] Li B, Zheng Y (2018). Dynamic evolution and phylogenomic analysis of the chloroplast genome in Schisandraceae. Sci. Rep..

[16] Nowicki M, Boggess SL, Saxton AM, Hadziabdic D, Xiang QJ, Molnar T, Huff ML, Staton ME, Zhao Y, Trigiano RN (2018). Haplotyping of *Cornus florida* and *C. kousa* chloroplasts: Insights into species-level differences and patterns of plastic DNA variation in cultivars. PLoS ONE.

[17] Zhang H, Hall N, Goertzen LR, Chen CY, Peatman E, Patel J, McElroy JS (2019). Transcriptome analysis reveals unique relationships among *Eleusine* species and heritage of *Eleusine coracana*. G3 (Bethesda).

[18] Wei R, Zhang XC (2020). Phylogeny of Diplazium (Athyriaceae) revisited: Resolving the backbone relationships based on plastid genomes and phylogenetic tree space analysis. Mol. Phylogenet. Evol..

[19] Mower JP (2009). The PREP suite: Predictive RNA editors for plant mitochondrial genes, chloroplast genes and user-defined alignments. Nucleic Acids Res..

[20] Li X, Tan W, Sun J, Du J, Zheng C, Tian X, Zheng M, Xiang B, Wang Y (2019). Comparison of four complete chloroplast genomes of medicinal and ornamental meconopsis species: Genome organization and species discrimination. Sci. Rep..

[21] Henriquez CL, Abdullah AI, Carlsen MM, Zuluaga A, Croat TB, McKain MR (2020). Molecular evolution of chloroplast genomes in Monsteroideae (Araceae). Planta.

[22] Xu F, He L, Gao S, Su Y, Li F, Xu L (2019). Comparative analysis of two sugarcane ancestors *Saccharum officinarum* and *S. spontaneum* based on complete chloroplast genome sequences and photosynthetic ability in cold stress. Int. J. Mol. Sci..

[23] Yan X, Liu T, Yuan X, Xu Y, Yan H, Hao G (2019). Chloroplast genomes and comparative analyses among thirteen taxa within Myrsinaceae s.str. clade (Myrsinoideae, Primulaceae). Int. J. Mol. Sci..

[24] Moner AM, Furtado A, Henry RJ (2020). Two divergent chloroplast genome sequence clades captured in the domesticated rice gene pool may have significance for rice production. BMC Plant Biol..

[25] Feiz L, Asakura Y, Mao L, Strickler SR, Fei Z, Rojas M, Barkan A, Stern DB (2020). CFM1, a member of the CRM-domain protein family, functions in chloroplast group II intron splicing in *Setaria viridis*. Plant J..

[26] Nellaepalli S, Ozawa SI, Kuroda H, Takahashi Y (2018). The photosystem I assembly apparatus consisting of Ycf3-Y3IP1 and Ycf4 modules. Nat. Commun..

[27] Liu H, Lu Y, Lan B, Xu J (2020). Codon usage by chloroplast gene is bias in *Hemiptelea davidii*. J. Genet..

[28] Cui N, Liao BS, Liang CL, Li SF, Zhang H, Xu J, Li XW, Chen SL (2020). Complete chloroplast genome of *Salvia plebeia*: Organization, specific barcode and phylogenetic analysis. Chin. J. Nat. Med..

[29] Wang S, Yang C, Zhao X, Chen S, Qu GZ (2018). Complete chloroplast genome sequence of *Betula platyphylla*: Gene organization, RNA editing, and comparative and phylogenetic analyses. BMC Genomics.

[30] Jiang W, Tan W, Gao H, Yu X, Zhang H, Bian Y, Wang Y, Tian X (2020). Transcriptome and complete chloroplast genome of *Glycyrrhiza inflata* and comparative analyses with the other two licorice species. Genomics.

[31] Chu D, Wei L (2020). Reduced C-to-U RNA editing rates might play a regulatory role in stress response of Arabidopsis. J. Plant Physiol..

[32] He P, Huang S, Xiao G, Zhang Y, Yu J (2016). Abundant RNA editing sites of chloroplast protein-coding genes in *Ginkgo biloba* and an evolutionary pattern analysis. BMC Plant Biol..

[33] Mader M, Pakull B, Blanc-Jolivet C, Paulini-Drewes M, Bouda ZH, Degen B, Small I, Kersten B (2018). Complete chloroplast genome sequences of four Meliaceae species and comparative analyses. Int. J. Mol. Sci..

[34] Wang Z, Weber JL, Zhong G, Tanksley SD (1994). Survey of plant short tandem DNA repeats. Theor. Appl. Genet..

[35] Lin WH, Kussell E (2012). Evolutionary pressures on simple sequence repeats in prokaryotic coding regions. Nucleic Acids Res..

[36] Lin E, Zhuang H, Yu J, Liu X, Huang H, Zhu M, Tong Z (2020). Genome survey of Chinese fir (*Cunninghamia lanceolata*): Identification of genomic SSRs and demonstration of their utility in genetic diversity analysis. Sci. Rep..

[37] Gomes Pacheco T, de Santana Lopes A, Monteiro Viana GD, Nascimento da Silva O, Morais da Silva G, do Nascimento Vieira L, Guerra MP, Nodari RO, Maltempi de Souza E, deOliveiraPedrosa F (2019). Genetic, evolutionary and phylogenetic aspects of the plastome of annatto (*Bixa orellana* L), the Amazonian commercial species of natural dyes. Planta.

[38] Duran C, Singhania R, Raman H, Batley J, Edwards D (2013). Predicting polymorphic EST-SSRs in silico. Mol. Ecol. Resour..

[39] Biju VC, Vijayan S, Rajan VS, Sasi A, Janardhanan A, Nair AS (2019). The complete chloroplast genome of *Trichopus zeylanicus*, and phylogenetic analysis with Dioscoreales. Plant Genome.

[40] Wang W, Messing J (2011). High-throughput sequencing of three Lemnoideae (duckweeds) chloroplast genomes from total DNA. PLoS ONE.

[41] Gao C, Deng Y, Wang J (1989). The complete chloroplast genomes of *Echinacanthus* species (Acanthaceae): Phylogenetic relationships, adaptive evolution, and screening of molecular markers. Front. Plant Sci..

[42] Jukes TH (2000). The neutral theory of molecular evolution. Genetics.

[43] de Boer HJ, Ichim MC, Newmaster SG (2015). DNA Barcoding and pharmacovigilance of herbal medicines. Drug Saf..

[44] Carbonell-Caballero J, Alonso R, Ibanez V, Terol J, Talon M, Dopazo J (2015). A phylogenetic analysis of 34 chloroplast genomes elucidates the relationships between wild and domestic species within the genus *Citrus*. Mol. Biol. Evol..

[45] Ma PF, Zhang YX, Zeng CX, Guo ZH, Li DZ (2014). Chloroplast phylogenomic analyses resolve deep-level relationships of an intractable bamboo tribe Arundinarieae (poaceae). Syst. Biol..

[46] Giannoulatou E, Park SH, Humphreys DT, Ho JW (2014). Verification and validation of bioinformatics software without a gold standard: A case study of BWA and Bowtie. BMC Bioinform..

[47] Xu GC, Xu TJ, Zhu R, Zhang Y, Li SQ, Wang HW, Li JT (2019). LR_Gapcloser: A tiling path-based gap closer that uses long reads to complete genome assembly. Gigascience.

[48] Tillich M, Lehwark P, Pellizzer T, Ulbricht-Jones ES, Fischer A, Bock R, Greiner S (2017). GeSeq—Versatile and accurate annotation of organelle genomes. Nucleic Acids Res..

[49] Lohse M, Drechsel O, Kahlau S, Bock R (2013). OrganellarGenomeDRAW—A suite of tools for generating physical maps of plastid and mitochondrial genomes and visualizing expression data sets. Nucleic Acids Res..

[50] Kumar S, Stecher G, Tamura K (2016). MEGA7: Molecular evolutionary genetics analysis version 7.0 for bigger datasets. Mol. Biol. Evol..

[51] Kurtz S, Choudhuri JV, Ohlebusch E, Schleiermacher C, Stoye J, Giegerich R (2001). REPuter: The manifold applications of repeat analysis on a genomic scale. Nucleic Acids Res..

[52] Martin DE (2006). The exact joint distribution of the sum of heads and apparent size statistics of a "tandem repeats finder" algorithm. Bull. Math. Biol..

[53] Hennequin C, Thierry A, Richard GF, Lecointre G, Nguyen HV, Gaillardin C, Dujon B (2001). Microsatellite typing as a new tool for identification of *Saccharomyces cerevisiae* strains. J. Clin. Microbiol..

[54] Mayor C, Brudno M, Schwartz JR, Poliakov A, Rubin EM, Frazer KA, Pachter LS, Dubchak I (2000). VISTA: Visualizing global DNA sequence alignments of arbitrary length. Bioinformatics.

[55] Katoh K, Standley DM (2013). MAFFT multiple sequence alignment software version 7: Improvements in performance and usability. Mol. Biol. Evol..

[56] Sun J, Wang Y, Liu Y, Xu C, Yuan Q, Guo L, Huang L (2020). Evolutionary and phylogenetic aspects of the chloroplast genome of Chaenomeles species. Sci. Rep..

