# Supplementary material for: Comparative genomic study on the complete plastomes of four officinal Ardisia species in China
Source: Sci Rep. 2021 Nov 15;11:22239. doi: 10.1038/s41598-021-01561-3 (PMC8594775; doi:10.1038/s41598-021-01561-3)
Supplement: Supplementary file 1 — Supplementary Tables. [file 41598_2021_1561_MOESM1_ESM.doc]

Comparative Genomic Study on the Complete Plastomes of

Four Officinal *Ardisia* Species

**Table S1: Genbank accession numbers of plastomes used in phylogenetic analysis of this work.**

| Accession number | Source species | Family |
| --- | --- | --- |
| NC_045111.1 | *Aegiceras corniculatum* | Primulaceae |
| NC_034641.1 | *Androsace bulleyana* |
| NC_039347.1 | *Androsace laxa* |
| NC_051991.1 | *Androsace mariae* |
| NC_034640.1 | *Androsace paxiana* |
| NC_045118.1 | *Tapeinosperma netor* |
| NC_045099.1 | *Elingamita johnsonii* |
| NC_045117.1 | *Tapeinosperma multiflorum* |
| NC_045100.1 | *Myrsine africana* |
| NC_045115.1 | *Parathesis chiapensis* |
| NC_045114.1 | *Parathesis donnell-smithii* |
| NC_045113.1 | *Embelia vestita* |
| NC_054287.1 | *Ardisia bullata* |
| KC465962.1 | *Ardisia polysticta* |
| NC_045098.1 | *Ardisia solanacea* |
| NC_053736.1 | *Lysimachia christinae* |
| NC_052863.1 | *Lysimachia hemsleyana* |
| NC_045116.1 | *Myrsine sandwicensis* |
| NC_045112.1 | *Myrsine stolonifera* |
| NC_046770.1 | *Primula beesiana* |
| NC_046947.1 | *Primula bulleyana* |
| MH394359.1 | *Primula chionantha* |
| NC_034678.1 | *Primula chrysochlora* |
| NC_050245.1 | *Primula chungensis* |
| NC_050247.1 | *Primula denticulata* |
| NC_051972.1 | *Primula filchnerae* |
| NC_039348.1 | *Primula handeliana* |
| NC_046771.1 | *Primula helodoxa* |
| NC_039350.1 | *Primula knuthiana* |
| NC_034371.1 | *Primula kwangtungensis* |
| KY235373.1 | *Primula matthioli* |
| NC_050244.1 | *Primula moupinensis* |
| NC_046415.1 | *Primula obconica* |
| NC_050848.1 | *Primula oreodoxa* |
| NC_050248.1 | *Primula pellucida* |
| NC_034331.1 | *Primula persimilis* |
| NC_024543.1 | *Primula poissonii* |
| NC_050246.1 | *Primula pulchella* |
| NC_050243.1 | *Primula sikkimensis* |
| NC_030609.1 | *Primula sinensis* |
| NC_034677.1 | *Primula stenodonta* |
| NC_046755.1 | *Primula tsiangii* |
| NC_031428.1 | *Primula veris* |
| NC_039349.1 | *Primula woodwardii* |
| NC_053764.1 | *Rhododendron simsii* | Ericaceae |

**Table S2: Gene annotated in the *Ardisia* complete chloroplast genomes.**

| Group of genes | Gene names |
| --- | --- |
| Photosystem I | *psaA, psaB, psaC, psaI, psaJ* |
| Photosystem Ⅱ | *psbA, psbB, psbC, psbD, psbE, psbF, psbH, psbI, psbJ, psbK, psbL, psbM, psbN, psbT, psbZ* |
| Cytochrome b/f complex | *petA, petB^*^, petD^*^, petG, petL, petN* |
| ATP synthase | *atpA, atpB, atpE, atpF^*^, atpH, atpI* |
| NADH dehydrogenase | *ndhA^*^, ndhB^*^*(×2)*, ndhC, ndhD, ndhE, ndhF, ndhG, ndhH, ndhI, ndhJ, ndhK* |
| RubisCO large submit | *rbcL* |
| RNA polymerase | *rpoA, rpoB, rpoC1^*^, rpoC2* |
| Ribosomal proteins(SSU) | *rps2, rps3, rps4, rps7*(×2)*, rps8, rps11, rps12^**^*(×2)*, rps14, rps15, rps16^*^, rps18, rps19* |
| Ribosomal proteins(LSU) | *rpl2^*^*(×2)*, rpl14, rpl16^*^, rpl20, rpl22, rpl23*(×2)*, rpl32, rpl33, rpl36* |
| Unknown function protein-coding gene | *ycf1*(×2)*, ycf2*(×2)*, ycf3^**^, ycf4* |
| Other genes | *accD, clpP^**^, matK, ccsA, cemA, infA* |
| Ribosomal RNAs | *rrn4.5*(×2)*, rrn5*(×2)*, rrn16*(×2)*, rrn23*(×2) |
| Transfer RNAs | *trnH-GUG, trnK-UUU^*^, trnQ-UUG, trnS-GCU, trnG-UCC^*^, trnR-UCU, trnC-GCA, trnD-GUC, trnY-GUA, trnE-UUC, trnT-GGU, trnS-UGA, trnG-GCC, trnA-FME, trnS-GGA, trnT-UGU, trnL-UAA^*^, trnF-GAA, trnV-UAC^*^, trnM-CAU*(×2)*, trnW-CCA, trnP-UGG, trnI-CAU, trnL-CAA*(×2)*, trnV-GAC*(×2)*, trnI-GAU^*^*(×2)*, trnA-UGC^*^*(×2)*, trnR-ACG*(×2)*, trnN-GUU*(×2)*, trnL-UAG* |

**Table S3:** Comparision of the lengths of introns from 18 intron-containing genes among four chloroplast genoms.

| Gene | species | Location | ExonⅠ（bp） | IntronⅠ（bp） | ExonⅡ（bp） | IntronⅡ（bp） | ExonⅢ（bp） |
| --- | --- | --- | --- | --- | --- | --- | --- |
| *trnK-UUU* | *A. gigantifolia* | LSC | 35 | 2534 | 37 | - | - |
|  | *A. crenata* |  | 35 | 2550 | 37 | - | - |
|  | *A. mamillata* |  | 35 | 2547 | 37 | - | - |
|  | *A. villosa* |  | 35 | 2547 | 37 | - | - |
| *rps16* | *A. gigantifolia* | LSC | 177 | 851 | 42 | - | - |
|  | *A. crenata* |  | 225 | 851 | 42 | - | - |
|  | *A. mamillata* |  | 225 | 848 | 42 | - | - |
|  | *A. villosa* |  | 225 | 849 | 42 | - | - |
| *trnG-UCC* | *A. gigantifolia* | LSC | 31 | 679 | 60 | - | - |
|  | *A. crenata* |  | 23 | 698 | 48 | - | - |
|  | *A. mamillata* |  | 23 | 693 | 48 | - | - |
|  | *A. villosa* |  | 23 | 693 | 48 | - | - |
| *atpF* | *A. gigantifolia* | LSC | 415 | 693 | 155 | - | - |
|  | *A. crenata* |  | 471 | 641 | 159 | - | - |
|  | *A. mamillata* |  | 471 | 635 | 159 | - | - |
|  | *A. villosa* |  | 471 | 635 | 159 | - | - |
| *rpoC1* | *A. gigantifolia* | LSC | 1626 | 752 | 435 | - | - |
|  | *A. crenata* |  | 1626 | 764 | 435 | - | - |
|  | *A. mamillata* |  | 1626 | 749 | 435 | - | - |
|  | *A. villosa* |  | 1620 | 751 | 435 | - | - |
| *ycf3* | *A. gigantifolia* | LSC | 155 | 1021 | 226 | 715 | 129 |
|  | *A. crenata* |  | 153 | 729 | 228 | 713 | 129 |
|  | *A. mamillata* |  | 155 | 729 | 226 | 713 | 129 |
|  | *A. villosa* |  | 155 | 729 | 226 | 712 | 129 |
| *trnL-UAA* | *A. gigantifolia* | LSC | 35 | 499 | 50 | - | - |
|  | *A. crenata* |  | 37 | 497 | 50 | - | - |
|  | *A. mamillata* |  | 37 | 507 | 50 | - | - |
|  | *A. villosa* |  | 37 | 507 | 50 | - | - |
| *trnV-UAC* | *A. gigantifolia* | LSC | 30 | 580 | 39 | - | - |
|  | *A. crenata* |  | 57 | 571 | 39 | - | - |
|  | *A. mamillata* |  | 37 | 581 | 39 | - | - |
|  | *A. villosa* |  | 37 | 581 | 39 | - | - |
| *rps12* | *A. gigantifolia* | LSC-IRs | 114 | - | 231 | 538 | 27 |
|  | *A. crenata* |  | 114 | - | 231 | 538 | 27 |
|  | *A. mamillata* |  | 114 | - | 231 | 538 | 27 |
|  | *A. villosa* |  | 114 | - | 231 | 538 | 27 |
| *clpP* | *A. gigantifolia* | LSC | 228 | 654 | 291 | 787 | 69 |
|  | *A. crenata* |  | 228 | 668 | 291 | 871 | 69 |
|  | *A. mamillata* |  | 228 | 657 | 291 | 877 | 69 |
|  | *A. villosa* |  | 228 | 657 | 291 | 876 | 69 |
| *petB* | *A. gigantifolia* | LSC | 6 | 761 | 642 | - | - |
|  | *A. crenata* |  | 6 | 760 | 642 | - | - |
|  | *A. mamillata* |  | 6 | 760 | 642 | - | - |
|  | *A. villosa* |  | 6 | 760 | 642 | - | - |
| *petD* | *A. gigantifolia* | LSC | 6 | 826 | 477 | - | - |
|  | *A. crenata* |  | 22 | 803 | 477 | - | - |
|  | *A. mamillata* |  | 6 | 833 | 477 | - | - |
|  | *A. villosa* |  | 6 | 833 | 477 | - | - |
| *rpl16* | *A. gigantifolia* | LSC | 399 | 996 | 9 | - | - |
|  | *A. crenata* |  | 399 | 997 | 9 | - | - |
|  | *A. mamillata* |  | 399 | 997 | 9 | - | - |
|  | *A. villosa* |  | 399 | 997 | 9 | - | - |
| *rpl2* | *A. gigantifolia* | IR | 435 | 652 | 393 | - | - |
|  | *A. crenata* |  | 435 | 652 | 393 | - | - |
|  | *A. mamillata* |  | 434 | 655 | 391 | - | - |
|  | *A. villosa* |  | 434 | 655 | 391 | - | - |
| *ndhB* | *A. gigantifolia* | IR | 756 | 679 | 777 | - | - |
|  | *A. crenata* |  | 756 | 679 | 777 | - | - |
|  | *A. mamillata* |  | 756 | 679 | 777 | - | - |
|  | *A. villosa* |  | 756 | 679 | 777 | - | - |
| *trnI-GAU* | *A. gigantifolia* | IR | 32 | 942 | 35 | - | - |
|  | *A. crenata* |  | 37 | 946 | 35 | - | - |
|  | *A. mamillata* |  | 37 | 945 | 35 | - | - |
|  | *A. villosa* |  | 37 | 944 | 35 | - | - |
| *trnA-UGC* | *A. gigantifolia* | IR | 37 | 813 | 36 | - | - |
|  | *A. crenata* |  | 38 | 813 | 35 | - | - |
|  | *A. mamillata* |  | 38 | 813 | 35 | - | - |
|  | *A. villosa* |  | 38 | 813 | 35 | - | - |
| *ndhA* | *A. gigantifolia* | SSC | 541 | 1087 | 551 | - | - |
|  | *A. crenata* |  | 540 | 1104 | 552 | - | - |
|  | *A. mamillata* |  | 541 | 1117 | 551 | - | - |
|  | *A. villosa* |  | 541 | 1087 | 551 | - | - |

**Table S4:** RSCU for protein-coding genes in the *Ardisia* plastomes.

| Amino acid | Codon | tRNA | *A. gigantifolia* | |  | *A. crenata* | |  | *A.mamillata* | |  | *A.villosa* | |
| --- | --- | --- | --- | --- | --- | --- | --- | --- | --- | --- | --- | --- | --- |
|  |  |  | Count | RSCU |  | Count | RSCU |  | Count | RSCU |  | Count | RSCU |
| Phe | UUU |  | 2268 | 1.24 |  | 2309 | 1.22 |  | 2321 | 1.22 |  | 2376 | 1.24 |
| Phe | UUC | *trnF-GAA* | 1384 | 0.76 |  | 1485 | 0.78 |  | 1479 | 0.78 |  | 1447 | 0.76 |
| Leu | UUA |  | 1192 | 1.31 |  | 1181 | 1.37 |  | 1094 | 1.27 |  | 1142 | 1.34 |
| Leu | UUG | *trnL-CAA* | 1055 | 1.16 |  | 1048 | 1.22 |  | 1027 | 1.19 |  | 1037 | 1.21 |
| Leu | CUU |  | 1149 | 1.27 |  | 1019 | 1.18 |  | 1107 | 1.29 |  | 1070 | 1.25 |
| Leu | CUC |  | 663 | 0.73 |  | 629 | 0.73 |  | 705 | 0.82 |  | 644 | 0.75 |
| Leu | CUA |  | 866 | 0.96 |  | 796 | 0.93 |  | 730 | 0.85 |  | 766 | 0.9 |
| Leu | CUG |  | 514 | 0.57 |  | 489 | 0.57 |  | 502 | 0.58 |  | 469 | 0.55 |
| Ile | AUU |  | 1736 | 1.22 |  | 1899 | 1.28 |  | 1830 | 1.26 |  | 1822 | 1.27 |
| Ile | AUC | *trnI-CAU* | 1052 | 0.74 |  | 1051 | 0.71 |  | 1080 | 0.72 |  | 978 | 0.68 |
| Ile | AUA |  | 1479 | 1.04 |  | 1517 | 1.02 |  | 1441 | 1.02 |  | 1516 | 1.05 |
| Met | AUG | *trnM-CAU* | 834 | 1 |  | 839 | 1 |  | 837 | 1 |  | 832 | 1 |
| Val | GUU |  | 822 | 1.42 |  | 834 | 1.4 |  | 809 | 1.46 |  | 769 | 1.36 |
| Val | GUC | *trnV-GAC* | 420 | 0.72 |  | 419 | 0.7 |  | 437 | 0.79 |  | 428 | 0.75 |
| Val | GUA |  | 683 | 1.18 |  | 752 | 1.26 |  | 615 | 1.11 |  | 671 | 1.18 |
| Val | GUG |  | 396 | 0.68 |  | 381 | 0.64 |  | 363 | 0.65 |  | 401 | 0.71 |
| Ser | UCU |  | 1178 | 1.52 |  | 1241 | 1.55 |  | 1187 | 1.49 |  | 1232 | 1.5 |
| Ser | UCC | *trnS-GGA* | 792 | 1.02 |  | 864 | 1.08 |  | 821 | 1.03 |  | 865 | 1.05 |
| Ser | UCA | *trnS-UGA,* | 991 | 1.28 |  | 963 | 1.2 |  | 975 | 1.22 |  | 990 | 1.2 |
| Ser | UCG |  | 628 | 0.81 |  | 566 | 0.71 |  | 639 | 0.8 |  | 595 | 0.72 |
| Pro | CCU |  | 682 | 1.09 |  | 707 | 1.15 |  | 649 | 1.07 |  | 649 | 1.07 |
| Pro | CCC |  | 613 | 0.98 |  | 609 | 0.99 |  | 588 | 0.97 |  | 614 | 1.01 |
| Pro | CCA | *trnP-UGG* | 765 | 1.23 |  | 764 | 1.24 |  | 793 | 1.31 |  | 765 | 1.26 |
| Pro | CCG |  | 432 | 0.69 |  | 387 | 0.63 |  | 389 | 0.64 |  | 396 | 0.65 |
| Thr | ACU |  | 619 | 1.12 |  | 713 | 1.22 |  | 679 | 1.2 |  | 679 | 1.15 |
| Thr | ACC | *trnT-GGU* | 538 | 0.97 |  | 567 | 0.97 |  | 560 | 0.99 |  | 605 | 1.02 |
| Thr | ACA | *trnT-UGU* | 697 | 1.26 |  | 714 | 1.22 |  | 661 | 1.17 |  | 711 | 1.2 |
| Thr | ACG |  | 362 | 0.65 |  | 351 | 0.6 |  | 368 | 0.65 |  | 371 | 0.63 |
| Ala | GCU |  | 452 | 1.18 |  | 552 | 1.34 |  | 476 | 1.29 |  | 485 | 1.26 |
| Ala | GCC |  | 361 | 0.94 |  | 375 | 0.91 |  | 375 | 1.02 |  | 401 | 1.04 |
| Ala | GCA | *trnA-UGC* | 451 | 1.18 |  | 483 | 1.18 |  | 406 | 1.1 |  | 433 | 1.13 |
| Ala | GCG |  | 269 | 0.7 |  | 234 | 0.57 |  | 218 | 0.59 |  | 219 | 0.57 |
| Tyr | UAU |  | 1562 | 1.37 |  | 1346 | 1.35 |  | 1490 | 1.36 |  | 1429 | 1.35 |
| Tyr | UAC | *trnY-GUA* | 714 | 0.63 |  | 651 | 0.65 |  | 702 | 0.64 |  | 687 | 0.65 |
| Stop | UAA |  | 1252 | 1.2 |  | 1155 | 1.19 |  | 1358 | 1.23 |  | 1282 | 1.25 |
| Stop | UAG |  | 876 | 0.84 |  | 731 | 0.75 |  | 865 | 0.78 |  | 748 | 0.73 |
| His | CAU |  | 998 | 1.44 |  | 909 | 1.42 |  | 972 | 1.45 |  | 938 | 1.44 |
| His | CAC | *trnH-GUG* | 385 | 0.56 |  | 372 | 0.58 |  | 370 | 0.55 |  | 368 | 0.56 |
| Gln | CAA | *trnQ-UUG* | 1098 | 1.39 |  | 1027 | 1.41 |  | 1088 | 1.39 |  | 1004 | 1.43 |
| Gln | CAG |  | 481 | 0.61 |  | 433 | 0.59 |  | 479 | 0.61 |  | 397 | 0.57 |
| Asn | AAU |  | 1699 | 1.35 |  | 1810 | 1.42 |  | 1772 | 1.41 |  | 1845 | 1.39 |
| Asn | AAC | *trnN-GUU* | 811 | 0.65 |  | 743 | 0.58 |  | 747 | 0.59 |  | 819 | 0.61 |
| Lys | AAA | *trnK-UUU* | 2181 | 1.35 |  | 2299 | 1.38 |  | 2186 | 1.36 |  | 2176 | 1.37 |
| Lys | AAG |  | 1047 | 0.65 |  | 1028 | 0.62 |  | 1018 | 0.64 |  | 1006 | 0.63 |
| Asp | GAU |  | 1072 | 1.43 |  | 1039 | 1.45 |  | 933 | 1.39 |  | 1038 | 1.46 |
| Asp | GAC | *trnD-GUC* | 428 | 0.57 |  | 397 | 0.55 |  | 405 | 0.61 |  | 381 | 0.54 |
| Glu | GAA | *trnE-UUC* | 1337 | 1.35 |  | 1377 | 1.41 |  | 1415 | 1.42 |  | 1295 | 1.38 |
| Glu | GAG |  | 645 | 0.65 |  | 581 | 0.59 |  | 580 | 0.58 |  | 578 | 0.62 |
| Cys | UGU |  | 693 | 1.25 |  | 642 | 1.16 |  | 737 | 1.21 |  | 704 | 1.16 |
| Cys | UGC | *trnC-GCA* | 418 | 0.75 |  | 465 | 0.84 |  | 484 | 0.79 |  | 509 | 0.84 |
| Stop | UGA |  | 1011 | 0.97 |  | 1033 | 1.06 |  | 1091 | 0.99 |  | 1035 | 1.01 |
| Trp | UGG | *trnW-CCA* | 665 | 1 |  | 669 | 1 |  | 677 | 1 |  | 662 | 1 |
| Arg | CGU | *trnR-ACG* | 356 | 0.67 |  | 388 | 0.71 |  | 366 | 0.67 |  | 376 | 0.67 |
| Arg | CGC |  | 262 | 0.5 |  | 261 | 0.48 |  | 260 | 0.48 |  | 261 | 0.46 |
| Arg | CGA |  | 568 | 1.07 |  | 583 | 1.07 |  | 606 | 1.11 |  | 633 | 1.13 |
| Arg | CGG |  | 387 | 0.73 |  | 356 | 0.66 |  | 364 | 0.67 |  | 334 | 0.59 |
| Ser | AGU |  | 621 | 0.8 |  | 695 | 0.87 |  | 672 | 0.84 |  | 714 | 0.87 |
| Ser | AGC |  | 448 | 0.58 |  | 482 | 0.6 |  | 499 | 0.62 |  | 546 | 0.66 |
| Arg | AGA | *trnR-UCU* | 1021 | 1.93 |  | 1079 | 1.99 |  | 1096 | 2.01 |  | 1143 | 2.03 |
| Arg | AGG | *trnS-GCU* | 581 | 1.1 |  | 593 | 1.09 |  | 581 | 1.07 |  | 629 | 1.12 |
| Gly | GGU |  | 538 | 0.95 |  | 606 | 1.05 |  | 550 | 0.97 |  | 574 | 1 |
| Gly | GGC | *trnG-GCC* | 371 | 0.66 |  | 348 | 0.6 |  | 322 | 0.57 |  | 356 | 0.62 |
| Gly | GGA | *trnG-UCC* | 772 | 1.37 |  | 818 | 1.42 |  | 840 | 1.48 |  | 805 | 1.4 |
| Gly | GGG |  | 576 | 1.02 |  | 529 | 0.92 |  | 558 | 0.98 |  | 565 | 0.98 |
| Total |  |  | 52,217 |  |  | 52,183 |  |  | 52,244 |  |  | 52,215 |  |

**Table S5:** Prediction of RNA editing sites in the plastomes of *Ardisia* species by the PREP program.

| Gene | Nucleotide position | | | | Condon (amino acid) change | Score |
| --- | --- | --- | --- | --- | --- | --- |
|  | *A. gigantifolia* | *A. crenata* | *A.mamillata* | *A.villosa* |  |  |
| *accD* | 1201 | 1201 | 1201 | 1201 | CCA (P) => TCA (S) | 1 |
|  | 1409 | 1409 | 1409 | 1409 | CCT (P) => CTT (L) | 1 |
| *atpA* | 914 | 914 | 914 | 914 | TCA (S) => TTA (L) | 1 |
| *atpB* | 35 | 35 | 35 | 35 | GCT (A) => GTT (V) | 0.86 |
| *atpF* | 92 | 92 | 92 | 92 | CCA (P) => CTA (L) | 0.86 |
| *matK* | 472 | 460 | 460 | 460 | CAC (H) => TAC (Y) | 1 |
|  | 703 | 691 | 691 | 691 | CCT (P) => TCT (S) | 1 |
|  | 1003 | 991 | 991 | 991 | CTT (L) => TTT (F) | 0.86 |
| *ndhA* | 341 | 341 | 341 | 341 | TCA (S) => TTA (L) | 1 |
|  | 566 | 566 | 566 | 566 | TCA (S) => TTA (L) | 1 |
|  | 1073 | 1073 | 1073 | 1073 | TCC (S) => TTC (F) | 1 |
| *ndhB* | 149 | 149 | 149 | 149 | TCA (S) => TTA (L) | 1 |
|  | 586 | 586 | 586 | 586 | CAT (H) => TAT (Y) | 1 |
|  | 746 | 746 | 746 | 746 | TCT (S) => TTT (F) | 1 |
|  | 830 | 830 | 830 | 830 | TCA (S) => TTA (L) | 1 |
|  | 1481 | 1481 | 1481 | 1481 | CCA (P) => CTA (L) | 1 |
| *ndhD* | 20 | 2 | 2 | 2 | ACG (T) => ATG (M) | 1 |
|  | 692 | 674 | 674 | 674 | TCG (S) => TTG (L) | 1 |
|  | 1094 | 1076 | 1076 | 1076 | GCT (A) => GTT (V) | 1 |
| *ndhF* | 253 | 253 | 253 | 253 | CTC (L) => TTC (F) | 1 |
|  | 1414 | 1414 | 1414 | 1414 | CAT (H) => TAT (Y) | 1 |
|  | 2168 | 2168 | 2168 | 2168 | TCA (S) => TTA (L) | 1 |
| *ndhG* | 166 | 166 | 166 | 166 | CAC (H) => TAC (Y) | 0.8 |
| *petB* | 611 | 611 | 611 | 611 | CCA (P) => CTA (L) | 1 |
| *psbB* | - | 370 | 370 | 370 | CGT (R) => TGT (C) | 1 |
| *psbE* | 214 | 214 | 214 | 214 | CCT (P) => TCT (S) | 1 |
| *psbF* | 77 | 77 | 77 | 77 | TCT (S) => TTT (F) | 1 |
| *rpoA* | 368 | 368 | 368 | 368 | TCA (S) => TTA (L) | 1 |
| *rpoB* | 211 | 211 | 211 | 211 | CAT (H) => TAT (Y) | 1 |
|  | 473 | 473 | 473 | 473 | TCA (S) => TTA (L) | 0.86 |
|  | 551 | 551 | 551 | 551 | TCA (S) => TTA (L) | 1 |
|  | 566 | 566 | 566 | 566 | TCG (S) => TTG (L) | 1 |
|  | 1360 | 1360 | 1360 | 1360 | CTT (L) => TTT (F) | 1 |
|  | 2336 | 2336 | 2336 | 2336 | ACA (T) => ATA (I) | 1 |
|  | 2782 | 2782 | 2782 | 2782 | CTT (L) => TTT (F) | 0.86 |
| *rpoC1* | 41 | 41 | 41 | 41 | TCA (S) => TTA (L) | 1 |
|  | 1967 | 1967 | 1967 | 1967 | ACA (T) => ATA (I) | 1 |
| *rpoC2* | 755 | - | - | - | GCA (A) => GTA (V) | 1 |
|  | - | - | 32 | - | CCG (P) => CTG (L) | 1 |
|  | 1294 | 1294 | 1294 | 1294 | CCC (P) => TCC (S) | 0.86 |
|  | 1456 | - | - | - | CGC (R) => TGC (C) | 0.86 |
|  | 1505 | 1505 | 1505 | 1505 | ACG (T) => ATG (M) | 0.86 |
|  | 2000 | - | - | - | GCA (A) => GTA (V) | 1 |
|  | - | - | 2008 | - | CTT (L) => TTT (F) | 1 |
|  | 2308 | 2308 | 2308 | 2308 | CGG (R) => TGG (W) | 1 |
|  | 2744 | 2744 | 2744 | 2744 | ACT (T) => ATT (I) | 1 |
|  | - | 2801 | 2801 | 2801 | ACT (T) => ATT (I) | 0.86 |
|  | 3146 | - | - | - | ACC (T) => ATC (I) | 0.86 |
|  | 3713 | 3713 | 3713 | 3713 | GCG (A) => GTG (V) | 1 |
|  | 3749 | 3749 | 3749 | 3749 | TCA (S) => TTA (L) | 0.86 |
| *rps8* | 182 | 182 | 182 | 182 | TCA (S) => TTA (L) | 0.86 |
| *rps14* | 80 | 80 | 80 | 80 | TCA (S) => TTA (L) | 1 |
|  | 257 | - | - | - | ACG (T) => ATG (M) | 1 |
| *rps2* | 248 | 248 | 248 | 248 | TCA (S) => TTA (L) | 1 |

**Table S6: Number of SSRs types among the *Ardisia* whole chloroplast genomes.**

| Type | Number of SSRs types | | | |
| --- | --- | --- | --- | --- |
|  | *A. gigantifolia* | *A. crenata* | *A.mamillata* | *A.villosa* |
| A | 61 | 66 | 61 | 64 |
| C | 2 | 3 | 2 | 2 |
| G | 1 | 1 | 1 | 1 |
| T | 86 | 86 | 87 | 87 |
| AG | 5 | 5 | 5 | 5 |
| AT | 12 | 13 | 11 | 11 |
| CT | 3 | 3 | 3 | 3 |
| GA | 3 | 4 | 4 | 4 |
| TA | 13 | 12 | 14 | 14 |
| TC | 4 | 4 | 4 | 4 |
| AAC | 2 | 2 | 2 | 2 |
| AAG | 3 | 3 | 3 | 3 |
| AAT | 1 | 3 | 2 | 2 |
| AGA | 4 | 4 | 3 | 3 |
| AGC | 1 | 1 | 1 | 1 |
| AGT | 1 | 1 | 1 | 1 |
| ATA | 5 | 5 | 6 | 6 |
| ATC | 1 | 1 | 1 | 1 |
| ATG | 1 | 1 | 1 | 1 |
| ATT | 7 | 8 | 6 | 6 |
| CAA | 1 | 1 | 1 | 1 |
| CAG | 1 | 1 | 1 | 1 |
| CTC | 1 | 1 | 1 | 1 |
| CTG | 1 | 1 | 1 | 1 |
| CTT | 3 | 3 | 3 | 3 |
| GAA | 2 | 2 | 2 | 2 |
| GAG | 1 | 1 | 1 | 1 |
| GAT | 1 | 1 | 1 | 1 |
| GCA | 1 | 1 | 1 | 1 |
| GCT | 1 | 1 | 1 | 1 |
| GGA | 1 | 1 | 1 | 1 |
| TAA | 4 | 4 | 4 | 4 |
| TAT | 4 | 4 | 4 | 4 |
| TCA | 1 | 1 | 1 | 1 |
| TCC | 1 | 1 | 1 | 5 |
| TCT | 4 | 4 | 5 | 2 |
| TGA | 2 | 2 | 2 | 1 |
| TGC | 1 | 1 | 1 | 1 |
| TTA | 5 | 4 | 4 | 4 |
| TTC | 6 | 6 | 6 | 6 |
| TTG | 3 | 3 | 3 | 3 |
| AAGA | - | 1 | 1 | 1 |
| AATA | 1 | 1 | 1 | 1 |
| ATAA | 1 | 1 | 1 | 1 |
| ATTT | 1 | 1 | 1 | 1 |
| GAAA | 1 | 1 | 1 | 1 |
| GAAT | 1 | 1 | 1 | 1 |
| TAGT | 1 | 1 | 1 | 1 |
| TTCT | 1 | 1 | 1 | 1 |
| TAAAT | - | 1 | 1 | - |
| TATAT | 1 | - | - | - |
| TATCTA | 1 | - | - | - |
